# Pharmaceuticals and Their Main Metabolites in Treated Sewage Sludge and Sludge-Amended Soil: Availability and Sorption Behaviour

**DOI:** 10.3390/molecules26195910

**Published:** 2021-09-29

**Authors:** Julia Martín, Carmen Mejías, Juan Luis Santos, Irene Aparicio, Esteban Alonso

**Affiliations:** Departamento de Química Analítica, Escuela Politécnica Superior, Universidad de Sevilla, C/Virgen de África, 7, E-41011 Seville, Spain; jbueno@us.es (J.M.); cmpadilla@us.es (C.M.); jlsantos@us.es (J.L.S.); iaparicio@us.es (I.A.)

**Keywords:** pharmaceuticals, metabolites, digested sludge, compost, soil, adsorption

## Abstract

This work evaluated the availability and sorption behaviour of four pharmaceuticals and eight of their metabolites in sewage sludge and sludge-amended soil. Digested sludge and compost were evaluated. The highest levels found in digested sludge corresponded to caffeine (up to 115 ng g^−1^ dm), ibuprofen (45 ng g^−1^ dm) and carbamazepine (9.3 ng g^−1^ dm). The concentrations measured in compost were even lower than in digested sludge. No compound was detected in sludge-amended soils. This fact could be due to the dilution effect after sludge application to soil. Different adsorption capacities in sludge–soil mixtures were measured for the studied compounds at the same spike concentration. In general, except for paraxanthine and 3-hydroxycarbamazepine, the metabolite concentrations measured in the mixtures were almost two-fold lower than those of their parent compounds, which can be explained by their mobility and lixiviation tendency. The log K_d_ ranged from −1.55 to 1.71 in sludge samples and from −0.29 to 1.18 in soil–sludge mixtures. The log K_d_ values calculated for compost were higher than those calculated for digested sludge. The obtained results implied that the higher organic carbon content of compost could influence soil contamination when it is applied to soil.

## 1. Introduction

Pharmaceutical compounds (PhCs) are essential products in our day-to-day life. After administration, most of the PhCs are partially metabolized and excreted as metabolites and/or parent compounds. Several works have reported poor removal rates of these compounds in wastewater treatment processes [1], and consequently, PhCs and their metabolites are released and detected in both effluent wastewater (usually up to μg L^−1^) and sewage sludge (from ng Kg^−1^ to mg kg^−1^) [2].

Currently, the application of sewage sludge in agricultural soils is largely encouraged for fertilising (due to its organic matter and nutrient content (N and P)) and conditioning, especially for overexploited lands [3]. Aerobically or anaerobically digested sludge, together with compost, are the main sludge products from wastewater treatment plants (WWTPs) applied to agricultural fields as fertilizers. According to the Spanish Sludge Registry, Spain is one of the EU countries with the highest values of sludge production (approximately 1,200,000 t dry matter (dm)/y), with the estimation that more than 50% of that amount is spread onto agricultural lands [4]. Despite the agronomic benefits of this practice, its safety is under discussion by the scientific community due to the contaminants, many of them of emerging as a concern, reported in sewage sludge [5]. PhCs and their metabolites present in soil can be subjected to adsorption/desorption, transport and/or degradation/transformation processes, which could introduce these compounds into the food chain and lead to potential human exposure [3,6,7].

Only a few studies have been focused on the influence and occurrence of pharmaceutical metabolites in soils. For example, recently, Malvar et al., (2021) [6] described the occurrence and degradation of some metabolites of pharmaceuticals and personal care products in Mediterranean soils. Transformations of parent compounds into their metabolites were observed only for ibuprofen (IBU), which was degraded to 2-hydroxyibuprofen (2-OH-IBU), whose concentration remained constant for 120 d in spite of the degradation of IBU. The authors observed in batch experiments a decrease with time of the concentrations in soils.

In addition to this degradation in soil, the adsorption/desorption process in sewage sludge has an important influence on the mobility of PhCs in the environment. Depending on these processes, sewage sludge can act both as a source or as a sink due to its adsorptive capacity [8]. The physicochemical properties of PhCs (log K_ow_, pK_a_) and soil properties (organic matter, clay content and pH) can play a main role in the mobility of PhCs in soil and, consequently, in soil adsorption, leaching, plant up-take and/or interaction with terrestrial organisms [3,8,9].

Data about the sorption/desorption behaviour of PhCs in sludge-amended soils are scarce. Most of the studies have been focused on the evaluation of the adsorption of parent compounds in soil [10,11]. These studies evaluated the adsorption of PhCs in soils, without considering their amendment, and were focused on a limited number of compounds, mainly antibiotics [12]. For example, Wang et al., (2015) [10] confirmed the increase of the sorption capacity of sulphonamides in soils amended with manure. The K_d_ values of sulphonamides in five different soils were in the range from 0.10 to 4.39, whereas the K_d_ values for five different soils amended with manure were 1.89 to 11.69. However, only a few studies have reported the sorption behaviour of the metabolites of these PhCs [7,13,14] despite these metabolites being present at higher concentrations and showing even higher toxicity than their parent compounds [14,15]. Moreover, most of the studies reported in the literature were based on batch experiments carried out by mixing the solid phase with a solution of the emerging pollutants. In addition, in most cases, single-compound systems were used, and the studies were mainly focused on carbamazepine and its metabolites [7,13,14].

The aim of this study was to investigate the availability of four PhCs, usually detected in sludge and soil samples, and their main metabolites in sewage sludge. Then, their sorption behaviour when agricultural soils are amended with sewage sludge was studied using batch experiments. Experiments were carried out by spiking solid samples with the target compounds. These assays were carried out using digested sludge and compost, in order to study the effect of both matrices in the retention and the concentration levels. The K_d_ values in sewage sludge and soil mixtures were calculated and evaluated. The studied PhCs were carbamazepine (CBZ), IBU, caffeine (CAF) and sulfamethoxazole (SMX) and nine of their metabolites (3-hydroxycarbamazepine (3-OH-CBZ), 10,11-dihydro-10-hydroxycarbamazepine (10-OH-CBZ), carbamazepine-10,11-epoxide (EP-CBZ), 1-hydroxyibuprofen (1-OH-IBU), 2-OH-IBU, carboxyibuprofen (CBX-IBU), 1,7-dimethylxanthine (PX), N^4^-acetylsulfamethoxazole (Ac-SMX)). This study covers the lack of experimental K_d_ data of metabolites of PhCs in sewage sludge, compost and soils amended with these compounds.

## 2. Results and Discussion

### 2.1. Concentrations of Pharmaceuticals and Their Metabolites in Digested Sludge, Compost and Soil Samples

Before the spiking procedure, six of the twelve selected compounds were detected in digested sludge with concentrations up to 115 ng g^−1^ dm, in the case of CAF. The concentration range, mean concentration and frequency of detection of each compound in digested sludge, compost and soil are summarized in Table 1.

The contents of 2-OH-IBU, CBX-IBU and 10-OH-CBZ metabolites in digested sludge were similar to those of their parent compounds. Globally, the lowest PhC concentrations were observed in compost samples. This concentration decrease from digested sludge to compost has been previously reported in the literature [16]. In that paper, the removal of micropollutants in compost ranged from 87% to 99%. This fact could be explained by the degradation of PhCs during the composting process, although other factors, such as the dilution effect caused by mixing sludge with other waste before composting, could contribute to this concentration decrease. The PhCs and their metabolites were not detected in any of the analysed soil samples.

CBZ was found in all samples at almost the same concentrations (7.77 ng g^−1^ dm in digested sludge and 2.77 ng g^−1^ dm in compost), as described by Bastos et al., (2020) [12], who reported concentrations of 224 ng g^−1^ dm in digested sludge and 112 ng g^−1^ dm in compost. Concentrations of 10-OH CBZ measured in digested sludge (9.4 ng g^−1^ dm) and compost (1.4 ng g^−1^ dm) were similar to those of its parent compound.

IBU was quantified in digested sludge at concentrations up to 45 ng g^−1^ dm. Higher or similar levels were found for two of its metabolites, CBX-IBU and 2-OH-IBU. This fact is in concordance with previous studies that described the formation of CBX-IBU, as the main degradation product of IBU by anaerobiosis [17]. The concentrations of IBU and its metabolites decreased to below the method quantification limits (<MQL) in compost samples, indicating a possible degradation under aerobic conditions. According to Malvar et al., (2020) [5], IBU concentrations decreased from 28.1 ng g^−1^ to 204 ng g^−1^ dm in mixed sludge and from 12.4 ng g^−1^ to 14.2 ng g^−1^ dm in aerobically digested and dehydrated sludge.

CAF showed significantly higher concentrations than PX in all digested sludge samples, whereas similar levels were reached in compost samples (18.1 ng g^−1^ dm and 12.7 ng g^−1^ dm for CAF and PX, respectively). Similar results were recently reported by Malvar et al., (2020) [5] in sewage sludge from the same geographic area, which could indicate a possible degradation of CAF into PX under aerobic conditions. SMX was the only PhC not detected. This result is consistent with other experiments at various scales, which have demonstrated its degradation by anaerobic digestion [9]. In spite of these results, the sorption behaviours of this compound and its main metabolite were evaluated. The concentrations of the studied compounds in soil:sludge and soil:compost mixtures at the beginning of the batch experiments were below the method detection limits.

### 2.2. Sorption Behaviour of Pharmaceuticals and Their Metabolites in Sludge-Amended Soil after Spiking

Sludge and compost were spiked with the target compounds to reach final concentrations from 1000 ng g^−1^ to 50,000 ng g^−1^. The soil:sludge mixtures were prepared by spiking sludge with the target compounds at different concentration levels to achieve a final concentration from 50 ng g^−1^ to 2500 ng g^−1^, as described in Section 3.2. Prior to the batch experiments, spiked sludge, compost and soil:sludge and soil:compost mixtures were analysed in order to control the possible loss of some of the studied compounds during the spiking procedure. The studied compounds were spiked at the same concentration levels, and the amount of contaminant adsorbed ranged from 29.7% (CAF) to 84.6% (CBZ) for most of the compounds (Table S3 in the Supplementary Materials). This fact could be due to the different physicochemical properties of the compounds and the characteristics of the evaluated sewage sludges [7]. CBZ and its metabolites were measured at concentration levels two-fold higher than those of the others selected compounds. Low amounts were adsorbed in the case of IBU and its metabolites. This fact could be due to the low retention of these compounds in soil and to their fast degradation, which could take place even during the spiking procedure [6]. This effect has been previously observed [6].

### 2.3. Phase Distribution of Pharmaceuticals and Their Metabolites in Different Solid Samples

**Figure 1:**
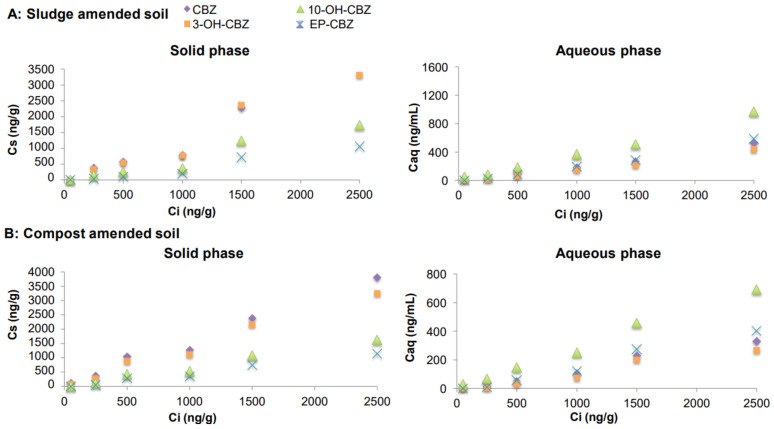
Concentrations of CBZ and its metabolites in soil samples amended with spiked sewage sludge (**A**) and compost (**B**).

**Figure 2:**
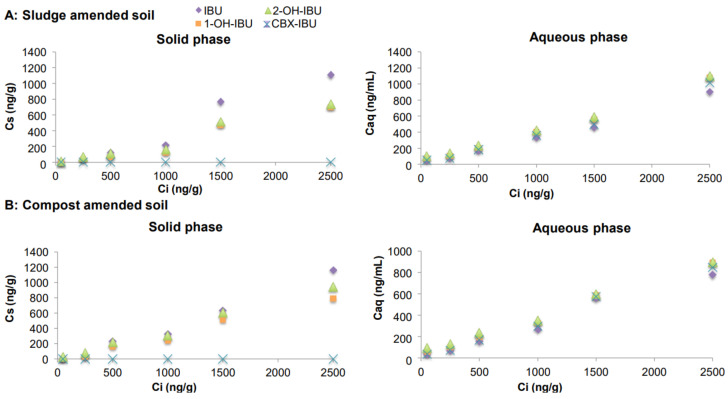
Concentrations of IBU and its metabolites in soil samples amended with spiked sewage sludge (**A**) and compost (**B**).

**Figure 3:**
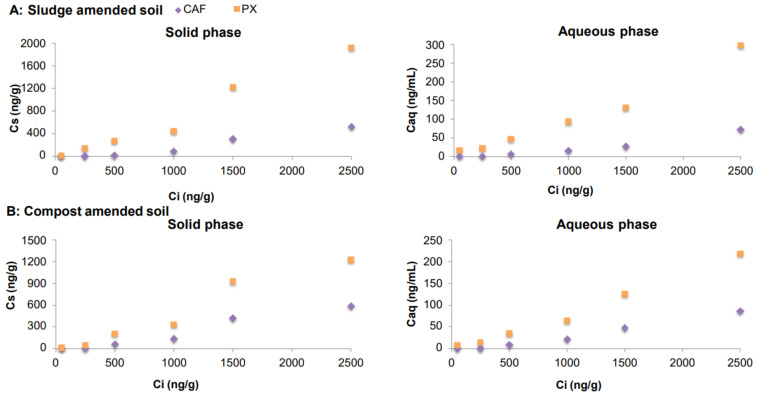
Concentrations of CAF and its metabolite in soil samples amended with spiked sewage sludge (**A**) and compost (**B**).

**Figure 4:**
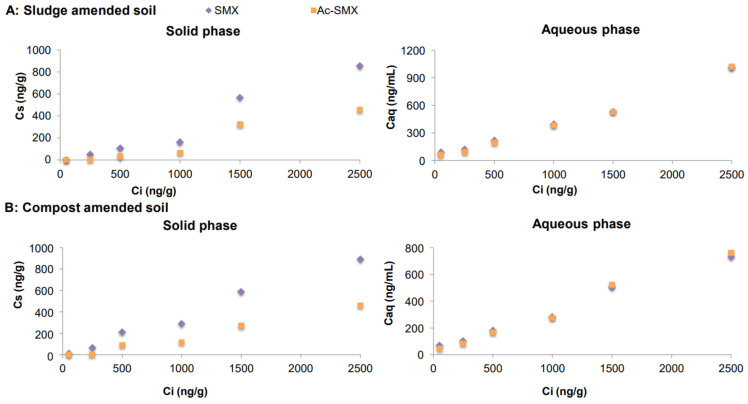
Concentrations of SMX and its metabolite in soil samples amended with spiked sewage sludge (**A**) and compost (**B**).

After batch experiments, the concentrations measured in soil-amended mixtures (C_s_) and water samples (C_aq_) in equilibrium (Y axis) and at the initial concentration (C_i_) (X axis) for CBZ, IBU, CAF and SMX and its metabolites are presented in Figures 1–4, respectively. The concentrations measured in digested sludge and compost samples and water samples are summarized in Table S4.

#### 2.3.1. Carbamazepine and Its Metabolites

CBZ was the PhC at the highest concentrations once sewage sludge was applied to soil. Although it was present in the water samples, its concentrations were low (from 11 ng g^−1^ to 526 ng g^−1^ in soil:sludge mixture and from 13 ng g^−1^ to 329 ng g^−1^ in soil:compost mixture). This fact could reflect an accumulation of this compound in amended soil, resulting in a very low mobility. The persistence of CBZ in sewage sludge has also been described in the literature. Malvar et al. (2020) [5] indicated that the concentrations of CBZ and 3-OH-CBZ only decreased slightly after aerobic and anaerobic treatments. The results were close to those published by Bourdat-Deschamps et al., (2017) [18], who considered CBZ as one of most persistent PhCs in soils. Chefetz et al., (2008) [11] also described a low mobility of CBZ in soils with high organic content.

A particular behaviour was observed for three of their metabolites (Figure 1). While 3-OH-CBZ presented a similar pattern to its parent compound, the concentrations of 10-OH-CBZ and EP-CBZ in the mixtures were almost two-fold lower than that of CBZ. This fact could be explained by their higher lixiviation tendency and their physicochemical properties. 3-OH-CBZ and CBZ present similar physicochemical properties and a higher log K_ow_ (2.4–2.5) than 10-OH-CBZ and EP-CBZ (0.9–1.0). Paz et al., (2016) [13] explained the different adsorption behaviours of CBZ, EP-CBZ and 10,11-dihydro−10,11-dihydroxycarbamazepine on the basis of their heterogeneous charge distribution density. EP-CBZ and 10-OH-CBZ present electronegative oxygen atoms in the epoxide and hydroxyl groups, respectively, which would allow forming hydrogen bonds with water molecules, inhibiting their adsorption onto the soil surface, whereas the close position of the hydroxyl group of 3-OH-CBZ to its amino group could contribute to the formation of hydrogen bonds with the organic matter of soil, increasing the stability of the molecule in soil [14]. Moreover, this effect was slightly more acute when digested sludge was applied to soil instead of compost (t_cal_ = 2.956, 3.787, 3.306 and 2.584 for CBZ, 3-OH-CBZ, 10-OH-CBZ and EP-CBZ, respectively t_tab_ = 2.571; *p* < 0.05).

#### 2.3.2. Ibuprofen and Its Metabolites

The concentrations of IBU, and especially of its metabolites, were higher in water samples once sewage sludge was applied to soil, indicating their higher leaching tendency and thus a high mobility in soil. In particular, CBX-IBU was not found in the mixtures, but it appeared in the water samples at similar concentrations to the other metabolites (Figure 2). Probably, part of this compound suffered degradation in sludge samples. The acidic compounds, characterized by low pK_a_ values, are expected to have a higher affinity for water because at environmental pH, they are mainly in their ionized form and, consequently, mainly dissolved in the aqueous phase. Similarly, the results from Lachassagne et al., (2015) [9] indicated that IBU was the PhC with the highest desorption potential for limed and digested sludge and the one most frequently found in amended soil leachates. Again, this effect was slightly more acute when digested sludge was applied to soil inside compost (t_cal_ = 2.584, 3.140, 2.905 and 3.163 for IBU, 1-OH-IBU, 2-OH-IBU and CBX-IBU, respectively; t_tab_ = 2.571; *p* < 0.05).

#### 2.3.3. Caffeine and Its Metabolite

In the case of CAF and PX, a different behaviour was observed once digested sludge or compost was applied to soil. The metabolite PX appeared at higher concentrations (more than double) than CAF (Figure 3). This fact could be due to a possible transformation of CAF into PX or to a higher retention of PX in soil. As the results reported by Greenham et al., (2019) [19], PX concentrations were higher than CAF concentrations. CAF is largely metabolized (<5% excreted unchanged in urine), so PX concentrations could be higher than CAF concentrations due to the high metabolizing rate.

#### 2.3.4. Sulfamethoxazole and Its Metabolite

The behaviour of SMX was opposed to that of CAF (Figure 4). When applied to soil, the Ac-SMX concentrations in the mixtures were almost two-fold lower than those of its parent compound. On the contrary, similar concentrations for both compounds were measured in water samples, indicating a higher leaching tendency of Ac-SMX. Similarly, the results reported by Andriamalala et al., (2018) [20] from incubation experiments using soil amended with organic waste indicated that Ac-SMX is slightly more mobile and mineralizable than SMX.

#### 2.3.5. Solid–Liquid Partition Coefficients

The K_d_ values obtained for digested sludge, compost and their mixtures with soil (5% *w*/*w*) calculated from the batch experiments are shown in Table 2. The log K_d_ values ranged from −1.55 (CBX-IBU) to 1.71 (3-OH-CBZ) in sludge samples and from −0.29 (Ac-SMX) to 1.18 (3-OH-CBZ) in soil mixtures. Overall, a clear decrease was observed in the K_d_ values when digested sludge or compost was applied to soil. The same pattern was observed for all the compounds. There is scarce information about the presence of metabolites in solid environmental samples [14]. Therefore, it was not possible to compare measured K_d_ data with other data in the literature. In addition, it is difficult to establish potential relations between the K_d_ values calculated under multiple and variable conditions and the physicochemical characteristics of sludge or compost. These statistical relations are generally weak [21,22].

Sorption occurs mainly through hydrophobic interactions between PhCs and organic matter in sludge [23]. PhCs with a moderate hydrophobic nature, such as CBZ, tend to partition into a particulate phase. A high correlation between the log K_d_ and log K_ow_ for CBZ and its metabolites was observed, as can be seen in Figure S1 (r^2^ > 0.71 and 0.85 in sewage sludge and in soil mixture, respectively; *p* < 0.05). Similarly, Wojsławski et al., (2019) [7] reported a lower K_d_ value (0.37) for 10-OH-CBZ in comparison to CBZ (1.87) in soil samples. The additional hydroxyl group significantly influences the hydrophobicity of the molecule, which is reflected in a reduction of the sorption capacity in the soil structure [13].

However, some of the PhCs and metabolites frequently detected in sludge are not lipophilic. PhCs containing polar functional groups can be retained on both organic and mineral fractions from soil and sludge. This fact could explain their sorption, regardless of their lipophilicity. High levels of hydrophilic compounds, such as CAF or PX (log K_ow_ −0.1 and −0.2) were found in this work. Moreover, PhCs can also be sorbed onto sludge by electrostatic interactions. Considering 2.9 as the pH of the isoelectric point of sludge [24], the surface of sludge will be negatively charged under typical environmental conditions (pH 5–8). Therefore, those PhCs existing in their neutral or positively charged forms at pH 5–8, such as CBZ, CAF and their metabolites, are expected to have higher sorption onto sewage sludge.

The physicochemical properties of PhCs are not the only factors determining their sorption behaviour onto sludge. Several authors have reported the formation of complexes between PhCs and their metabolites with metal cations (Mg^2+^, Ca^2+^ and Cu^2+^) in sludge, which could influence the sorption behaviour of the target compounds [35]. The organic carbon content of sludge is frequently cited as an important parameter determining the sorption of pharmaceuticals [23]. The log K_d_ values in compost samples were significantly higher than those calculated for digested sludge (t_cal_ = 5.616, 7.869 and t_tab_ = 2.201, 2.228; *p* < 0.05 for sewage sludge and soil mixtures, respectively). This fact could be due to a better sorption onto compost than onto digested sludge and/or to a fast leaching when sludge is applied to soil. Filipe et al., (2010) [36] also observed higher K_d_ values when soil was amended with compost in comparison to being amended with digested sludge and farmyard manure, which was the matrix with the highest organic carbon content. Recently, Albero et al., (2018) [3] and Wang et al., (2015) [10] observed an increase in the apparent sorption coefficients of antibiotics when soil was amended with compost. Mordechay et al., (2018) [8] described an increase in the bioavailability of CBZ present in sewage sludge when the soil organic carbon content that controls its sorption onto soils decreased.

Finally, the relatively low K_d_ values of IBU and SMX and their metabolites could be attributed to the moderate or high mobility of these compounds in soil.

## 3. Materials and Methods

### 3.1. Sewage Sludge and Soil

Digested sludge was sampled from a WWTP sited in Seville (Southern Spain). The digested sludge samples were collected from an anaerobic digester in which a mixture of primary and secondary sludge was treated together. Digested sludge was then dewatered by centrifugation. Compost sludge was collected from a composting plant where digested sludge from four WWTPs sited in Seville was composted in dynamic batteries with aeration facilitated by turning. Amounts of 5 kg of anaerobically digested and dewatered sludge and 5 kg of compost sludge were collected for the experiments. The Mediterranean agricultural soil was collected from Seville (Spain). It was a Cambisol soil, frequently found in European countries such as Italy, Greece, Germany, France and Spain. An amount of 5 kg of surface soil (0–20 cm) was sampled and homogenized.

All matrices were lyophilized in a Cryodos-50 lyophilizer (Telstar, Terrasa, Spain), sieved (particle size <100 μm), stored in amber glass bottles and maintained at −18 °C before the experiments started. PhCs and their metabolites were analysed in nonspiked samples before batch experiments in order to consider the previous concentrations of each compound.

### 3.2. Batch Experiments

Experiments were carried out using four types of solid samples: digested sludge, compost and soil amended with digested sludge or with compost (5% *w*/*w*). First, the initial concentrations of the target compounds in all solid samples were measured. Taking into account the concentrations of PhCs and their metabolites expected in sludge samples [5,37], digested sludge and compost were spiked with the target compounds to achieve a final concentration from 1000 ng g^−1^ to 50,000 ng g^−1^. The soil:sludge mixtures were prepared by spiking sludge with the target compounds at different concentration levels to achieve a final concentration from 50 ng g^−1^ to 2500 ng g ^−1^. The standard solutions used for spiking were prepared in water, adding, when necessary, the lowest volume as possible of methanol. Then, the spiked sludge sample was lyophilized, sieved and mixed with nonspiked soil. The final spike concentrations in all solid samples were measured in order to know the sorption behaviour of pharmaceuticals and their metabolites after the spiking procedure.

**Figure 5:**
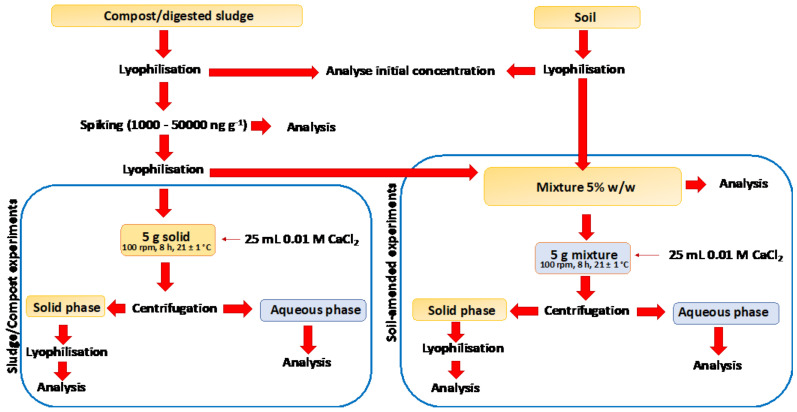
Schematic representation of the batch experiments.

Batch experiments were carried out according to the OECD guidelines [38] (Figure 5). The applied solid:water solution ratio was 1:5. For each solid type (sludge, compost, sludge amended soil and compost amended soil), six concentrations levels were prepared in triplicate. The solids were homogenized and distributed in centrifuge tubes (5 g of each matrix), and 25 mL of 0.01 M CaCl_2_ solution was added [36,38]. The centrifuge tubes were shaken on an end-over-end shaker at 100 rpm, during 8 h at 21 ± 1 °C, to achieve the equilibrium state. Then, the tubes were centrifuged at 4050× *g* for 10 min. The solid residue was freeze-dried (0.01 mbar vacuum and at −18 °C for 24 h), and the liquid phase was frozen until the analysis.

### 3.3. Analysis of the Pharmaceuticals and Metabolites

The analysis of the target compounds in the solid samples was carried out according to Malvar et al., (2020) [36]. The analysis of the target compounds in the liquid phase was carried out by filtering the liquid phase through a 0.2 μm cellulose syringe filter and measuring these compounds by direct injection into the LC-MS/MS system. Detailed information of the analytical method applied can be found in the Supplementary Materials (Tables S1 and S2).

### 3.4. Solid–Water Partition Coefficients (K_d_)

Solid–water partition coefficients (K_d_), corresponding to the distributions of PhCs and their metabolites between solid samples (digested sludge, compost and soil) and water were calculated as K_d_ = C_solid_/C_water_, where K_d_ is expressed in L kg ^−1^. C_solid_ refers to the concentrations of the target compounds in solid form in the equilibrium state, and C_water_ refers to the concentrations of the target compounds in water in the equilibrium state.

## 4. Conclusions

Two different organic waste products (digested sludge and compost) were used to amend agricultural soils in laboratory experiments simulating real conditions. The highest concentrations found in digested sludge corresponded to CAF, followed by IBU and CBZ. The concentrations detected in compost samples were very low, whereas the compounds were not detected in soil samples, indicating an important role of the dilution effect.

In the batch experiments, the sorption of the contaminants onto amended soil was dependent on their physicochemical properties and on the sewage sludge type. The percentage of the contaminants adsorbed ranged from 29.6% to 84.6%. CBZ was the compound with the highest adsorption onto sludge and the most persistent one, while low amounts were adsorbed in the case of IBU and its metabolites, which can be attributed to their low affinity for solid particles and also to their rapid degradation. Overall, a decrease in metabolite content was observed with the exception of CAF, which underwent a particular behaviour. Once sewage was applied to soil, its metabolite PX appeared at higher concentrations (more than double) than CAF.

The log K_d_ ranged from −1.55 (CBX-IBU) to 1.71 (3-OH-CBZ) in sludge samples and from −0.29 (Ac-SMX) to 1.18 (3-OH-CBZ) in sludge:soil mixtures. Overall, a clear decrease was observed in the log K_d_ values when digested sludge or compost were applied to soil, although the same pattern was observed for all the compounds. The relatively low K_d_ values of IBU, SMX and their metabolites indicated their moderate to high availability in soil. Moreover, log K_d_ values measured when compost was applied to soil were significantly higher than those calculated in digested sludge, indicating a possible influence of its higher organic carbon content.

The results obtained indicated that, despite the application of organic residues to improve agricultural soils possibly being a sustainable practice, their use as fertilizers should be done with caution. Moreover, the concentrations of PhCs and their metabolites measured in sludge and compost revealed the necessity to assess the potential ecotoxicological risks associated with their use as fertilizers.

## Figures and Tables

**Table 1:** Concentration levels (range (ng g^−1^ dm), mean (ng g^−1^ dm) and RSD (%)) of the selected PhCs and their metabolites in solid samples (*n* = 5).

Compound	Digested Sludge			Compost			Soil		
	Range	Mean	RSD	Range	Mean	RSD	Range	Mean	RSD
CBZ	6.7–9.3	7.77	1.36	1.4–2.8	2.17	0.71	n.d.	-	-
3-OH-CBZ	<MQL	-	-	<MQL-3.7	2.60	1.55	n.d.	-	-
10-OH-CBZ	6.0–16	9.40	5.72	n.d.-2.3	1.70	0.52	n.d.	-	-
EP-CBZ	n.d.	-	-	n.d.	-	-	n.d.	-	-
IBU	<MQL-45	29.8	14.3	<MQL	-	-	n.d.	-	-
1-OH IBU	<MQL	-	-	n.d.	-	-	n.d.	-	-
2-OH IBU	34–64	47.3	15.3	n.d.	-	-	n.d.	-	-
CBX-IBU	15–32	25.3	9.1	n.d.	-	-	n.d.	-	-
CAF	<MQL-115	50.1	19.8	<MQL-36	18.1	12.1	n.d.	-	-
PX	<MQL	-	-	<MQL-17	12.7	5.90	n.d.	-	-
SMX	n.d.	-	-	n.d.	-	-	n.d.	-	-
Ac-SMX	n.d.	-	-	n.d.	-	-	n.d.	-	-

<MQL: below the method quantification limit; n.d.: not detected.

**Table 2:** Physicochemical properties (log K_ow_ and pK_a_ values) and calculated log K_d_ values of the pharmaceutically active compounds.

**Compound**	**Physicochemical Properties**						**Literature log K_d_ Values**					
	**pK_a_**			**Log K_ow_**			**Sludge**			**Soil**		
CBZ	13.9 ^a^			2.5 ^b^			−1.59 ^h^–1.63 ^i^			0.22 ^j^–1.11 ^i^		
3-OH-CBZ	9.19 ^c^			2.41 ^d^			-- ^o^			0.20–1.00 ^j^		
10-OH-CBZ	12.8 ^d^			0.93 ^e^			-- ^o^			−0.85 ^j^–−0.43 ^k^		
EP-CBZ	16.0 ^d^			1.0 ^b^			-- ^o^			−0.40–0.00 ^j^		
IBU	4.9 ^b^			4.0 ^b^			1.49 ^h^			−0.20–0.10 ^l^		
1-OH-IBU	4.55 ^f^			2.69 ^f^			-- ^o^			-- ^o^		
2-OH-IBU	4.63 ^f^			2.37 ^f^			-- ^o^			-- ^o^		
CBX-IBU	3.97 ^f^			2.78 ^f^			-- ^o^			-- ^o^		
CAF	10.4 ^a^			−0.1 ^b^			1.14 ^h^			−0.39 ^m^–1.39 ^i^		
PX	8.5 ^a^			−0.2 ^b^			-- ^o^			-- ^o^		
SMX	5.7 ^b^			0.9 ^b^			1.39 ^h^			0.41–0.50 ^n^		
Ac-SMX	5.54 ^g^			1.18 ^g^			-- ^o^			0.00–0.12 ^n^		
**Calculated log K_d_ Values**												
**Compound**	**Sludge**			**Compost**			**Sludge:Soil**			**Compost:Soil**		
	**Mean**		**S**	**Mean**		**S**	**Mean**		**S**	**Mean**		**S**
CBZ	1.37	±	0.11	1.67	±	0.23	0.81	±	0.13	1.12	±	0.13
3-OH-CBZ	1.49	±	0.20	1.71	±	0.34	0.90	±	0.14	1.18	±	0.18
10-OH-CBZ	0.63	±	0.09	0.74	±	0.09	0.19	±	0.16	0.38	±	0.06
EP-CBZ	0.88	±	0.12	1.09	±	0.21	0.19	±	0.17	0.51	±	0.12
IBU	0.45	±	0.07	0.74	±	0.07	0.00	±	0.19	0.12	±	0.06
1-OH-IBU	0.34	±	0.13	0.22	±	0.09	−0.32	±	0.22	−0.08	±	0.04
2-OH-IBU	0.37	±	0.12	0.20	±	0.09	−0.25	±	0.16	−0.02	±	0.04
CBX-IBU	−1.50	±	0.31	−1.55	±	0.12	- ^p^			- ^p^		
CAF	1.60	±	0.21	1.06	±	0.15	0.74	±	0.33	0.85	±	0.06
PX	0.91	±	0.09	0.97	±	0.11	0.81	±	0.12	0.78	±	0.07
SMX	0.33	±	0.07	0.60	±	0.08	−0.18	±	0.19	0.06	±	0.03
Ac-SMX	0.27	±	0.10	0.49	±	0.08	−0.53	±	0.29	−0.29	±	0.07

^a^ Rosal et al., 2010 [25]; ^b^ Muñoz et al., 2008 [26]; ^c^ Lee et al., 2011 [27]; ^d^ Huntscha et al., 2012 [28]; ^e^ Miao et al., 2005 [29]; ^f^ Ferrando-Climent et al., 2012 [30]; ^g^ Baumer et al., 2017 [31]; ^h^ Berthod et al., 2016 [22]; ^i^ Barron et al., 2009 [32]; ^j^ Malvar et al., 2020 [14]; ^k^ Wojsławski et al., 2019 [7]; ^l^ Mitra and Benfield 2018 [33]; ^m^ Gielen et al., 2009 [34]; ^n^ Andriamalala et al., (2018) [20]; ^o^ values not found; ^p^ value not calculated.

## Data Availability

Not applicable.

## Supplementary Materials

Figure S1: Correlations between log K_d_ and log K_ow_ for CBZ and its metabolites in sewage sludge (top) and soil mixtures (bottom) (*n* = 42), Table S1: LC-MS/MS parameters, Table S2: Accuracy (%), precision, expressed as the relative standard deviation (%), extraction recovery, method detection limits (MDL), method quantitation limits (MQL) and the matrix effect, Table S3: Efficiency of the spike procedure of PhCs and their metabolites in sludge and compost, Table S4: Concentration of PhCs and metabolites measured in solid (C_s_) and aqueous (C_aq_) samples in the batch experiments.

## Sample Availability

Samples of the compounds are available from the authors.
