# Supplementary material for: Pharmaceuticals and Their Main Metabolites in Treated Sewage Sludge and Sludge-Amended Soil: Availability and Sorption Behaviour
Source: Molecules. 2021 Sep 29;26(19):5910. doi: 10.3390/molecules26195910 (PMC8513156; doi:10.3390/molecules26195910)
Supplement: Supplementary file 1 [file molecules-26-05910-s001.zip › molecules-1386076-supplementary.pdf]

molecules

Supplemetary Materials

Pharmaceuticals and Their Main Metabolites in Treated Sewage Sludge and Sludge-Amended Soil: Availability and Sorption Behaviour

Julia Martín, Carmen Mejías, Juan Luis Santos, Irene Aparicio and Esteban Alonso *

Departamento de Química Analítica, Escuela Politécnica Superior, Universidad de Sevilla. C/Virgen de África, 7, E–41011 Seville, Spain; jbueno@us.es (J.M.); cmpadilla@us.es (C.M.); jlsantos@us.es (J.L.S.); iaparicio@us.es (I.A.)
* Correspondence: ealonso@us.es

Figure captions:

Figure S1. Correlations between log K_d and log K_{ow} for CBZ and its metabolites in sewage sludge (up) and soil mixtures (bottom) (n=42).

Table captions:

Table S1. LC-MS/MS parameters.

Table S2. Accuracy (%), precision, expressed as relative standard deviation (%), extraction recovery, method detection limits (MDL) and method quantitation limits (MQL) and matrix effect.

Table S3. Efficiency of the spike procedure of PhCs and their metabolites on sludge and compost.

Table S4. Concentration of PhCs and metabolites measured in solid (C_s) and aqueous (C_{aq}) samples in batch experiments.

Analytical method

Chemicals and reagents

HPLC-grade methanol (MeOH) and water were supplied by Romil (Barcelona, Spain). Analytical-grade formic acid (98%) and calcium chloride were obtained from Panreac (Barcelona, Spain). Primary-secondary amine (PSA) and C18 were provided by Scharlab (Barcelona, Spain). High purity standards of selected PhCs and metabolites and the internal standards (IS) caffeine-$^{13}C_3$ (CAF-$^{13}C_3$), and ibuprofen-d_3 (IBU-d_3) were provided by Sigma-Aldrich (St. Louis MO, USA). Individual stock standard solutions were prepared at 1000 mg L^{-1} in MeOH and stored at -18 °C. Working solutions were prepared by dilution of the standard stock solutions in water.

Analytical determination

The analysis of studied compounds was based on ultrasonic assisted extraction and determination by high performance liquid chromatography-tandem-mass spectrometry. Briefly, aliquots of the samples (1 g) were weighed into 12 mL glass vials, containing 100 µL of a methanol solution (250 ng mL^{-1}) of a mixture of ISs. The samples were ultrasonicated three times using 5 mL of MeOH (0.5% formic acid) and centrifuged for 10 min at 4050 × g. For clean-up, dispersive solid phase extraction (d-SPE) was applied. The supernatants obtained were combined in a 50 mL polypropylene conical tube containing 400 mg of PSA and 800 mg C18 sorbents. The mixture was hand-shaken for 2 min and centrifuged for 5 min at 4050 × g. The solvent was evaporated to dryness at room temperature under a nitrogen stream, reconstituted in 0.5 mL of MeOH:water (50:50, v/v) and filtered

through a 0.22 µm nylon filter. A 10 µL aliquot of the extract was injected into the LC instrument.

Liquid chromatography-tandem mass spectrometry analysis was performed using a Kinetex® Polar C18 column (50 mm x 3.0 mm i.d., 2.6 µm particle size) (Phenomenex, Torrance CA) thermostated at 35 °C and protected by a SecurityGuardTM ULTRA C18 guard column (2 mm x 3 mm i.d.) (Phenomenex, Torrance CA). The compounds were separated using a gradient mobile phase consisting of water containing formic acid (0.1%, v/v) and MeOH containing formic acid (0.1%, v/v). Two multiple reaction monitoring (MRM) transitions were selected for each analyte for quantification and confirmation of compounds (Table S1).

The validation characteristics of the method (sensitivity, trueness and precision) are summarized in Table S2 and were previously reported by Malvar et al. (2020) [35]. For each batch of samples, samples, matrix-matched calibration standards, procedural blanks, and spiked samples were processed.

Table S1. LC-MS/MS parameters.

Compound	Ionization mode	Precursor ion (*m/z*)	Product ions (MRM1/MRM2) (*m/z*)	Fragmentor (V)	CE (V)	RT (min)
CBZ	Positive	237.1	194.1/179.0	140	16/36	13.2
3-OH-CBZ	Positive	253.1	210.1/167.0	120	16/40	11.4
10-OH-CBZ	Positive	255.1	194.1/237.1	60	20/4	10.3
EP-CBZ	Positive	253.1	180.1/236.1	60	24/4	10.7
IBU	Positive	207.1	161.1/119.1	100	4/20	21.4
1-OH-IBU	Negative	221.1	159.1/177.1	60	4/0	14.2
2-OH-IBU	Negative	221.1	177.1	40	0	12.7
CBX-IBU	Negative	235.1	191.1/73.0	40	0/8	13.2
CAF	Positive	195.1	42.1/138.0	100	40/16	6.4
PX	Positive	181.1	124.0/42.1	80	20/40	4.6
SMX	Positive	254.1	92.1/65.0	80	28/40	6.8
Ac-SMX	Positive	296.1	65.0/134.0	60	40/20	9.2

Table S2. Accuracy (%), precision, expressed as relative standard deviation (%), extraction recovery, method detection limits (MDL) and method quantitation limits (MQL) and matrix effect.

Compounds	Accuracy (%)			Precision (%)			R (%)	MDL (µg kg⁻¹ dm)	MQL (µg kg⁻¹ dm)	Matrix effect (%)
	Low level (25 µg kg^{-1})	Medium level (62.5 µg kg^{-1})	High level (125 µg kg^{-1})	Low level (25 µg kg^{-1})	Medium level (62.5 µg kg^{-1})	High level (125 µg kg^{-1})				
**CBZ**	**106**	**100**	**100**	**1.6**	**3.1**	**0.4**	**102**	**0.07**	**0.24**	-1
3-OH-CBZ	106	101	98	2.2	1.1	0.2	98	0.04	0.13	-6
10-OH-CBZ	101	100	97	2.3	1.0	0.1	99	0.04	0.12	1
EP-CBZ	105	103	101	1.0	1.8	0.1	86	0.04	0.14	-18
IBU	116	116	108	2.3	3.1	3.8	61	0.53	1.76	-6
1-OH-IBU	120	110	118	3.8	0.8	6.8	96	0.42	1.39	-2
2-OH-IBU	111	98	104	3.5	1.0	5.7	97	0.80	2.66	-3
CBX-IBU	111	100	105	3.2	6.0	8.9	77	0.51	1.69	-15
CAF	65	67	66	9.9	4.3	13	26	1.34	4.46	-2
PX	66	80	93	3.6	15	18	21	1.50	5.00	-7
SMX	97	113	111	6.1	6.3	3.5	36	0.30	1.00	-8
Ac-SMX	108	107	106	3.1	2.4	1.2	100	0.07	0.25	-4

Table S3. Efficiency of the spike procedure of PhCs and their metabolites on sludge and compost.

Compound	Spiking efficiency
CBZ	81.5
3-OH-CBZ	74.2
10-OH-CBZ	84.6
EP-CBZ	48.9
IBU	40.0
1-OH-IBU	66.2
2-OH-IBU	67.9
CBX-IBU	37.0
CAF	29.7
PX	38.2
SMX	63.4
Ac-SMX	60.0

Table S4. Concentration of PhCs and metabolites measured in solid (C_s) and aqueous (C_{aq}) samples in batch experiments.

Spiked Conc.	CBZ		3-OH-CBZ		10-OH-CBZ		EP-CBZ		IBU		1-OH-IBU		2-OH-IBU		CBX-IBU		CAF		PX		SMX		Ac-SMX	
	Cs	Caq	Cs	Caq	Cs	Caq	Cs	Caq	Cs	Caq	Cs	Caq	Cs	Caq	Cs	Caq	Cs	Caq	Cs	Caq	Cs	Caq	Cs	Caq
(ng g-1)	(ng g-1)	(ng mL-1)	(ng g-1)	(ng mL-1)	(ng g-1)	(ng mL-1)	(ng g-1)	(ng mL-1)	(ng g-1)	(ng mL-1)	(ng g-1)	(ng mL-1)	(ng g-1)	(ng mL-1)	(ng g-1)	(ng mL-1)	(ng g-1)	(ng mL-1)	(ng g-1)	(ng mL-1)	(ng g-1)	(ng mL-1)	(ng g-1)	(ng mL-1)
Sludge																								
1000	1515	43	1654	23	1259	208	794	63	308	96	656	184	690	198	17	155	370	2	471	45	500	208	482	181
5000	2993	120	3043	95	1780	448	1353	185	610	202	744	382	740	381	16	376	859	11	977	105	753	408	646	387
10000	6274	251	6084	192	3836	902	2906	368	1335	407	1855	810	1883	778	23	754	1837	38	2051	227	1712	764	1526	785
20000	9874	494	9139	430	4916	1501	4233	752	1997	763	2077	1403	2054	1299	22	1391	3915	94	3587	487	2108	1296	1794	1376
30000	14739	681	13775	471	9487	2231	7445	1043	3064	1166	5356	2255	5746	2190	54	2216	5023	177	5334	762	4794	1927	4384	2126
50000	21061	1235	19423	994	15032	3803	11561	1842	4636	2128	8652	4316	9749	4259	69	3869	9576	428	9160	1509	7702	3443	7020	3848
Compost																								
1000	1756	15	1577	7	889	133	756	26	393	70	284	190	301	212	<MDL	177	616	31	591	44	603	151	458	134
5000	2751	55	2333	42	1420	278	1119	92	817	155	576	376	554	403	15	377	839	78	810	102	952	278	803	280
10000	7085	130	5879	113	3583	549	3027	215	2201	316	1516	757	1424	786	24	762	2186	170	2137	211	2524	511	2085	556
20000	10085	278	8218	245	4910	1034	4233	452	3353	650	2160	1440	2027	1425	29	1368	3333	365	3578	443	3593	949	2988	1081
30000	15458	377	13373	338	8372	1317	7184	611	5326	847	4091	1836	4039	1826	55	1734	6417	493	6861	606	6028	1209	5028	1401
50000	18666	820	16029	761	10386	2620	9030	1327	7153	1642	4851	3650	4797	3576	71	3366	8020	1086	8999	1332	7626	2355	6484	2833

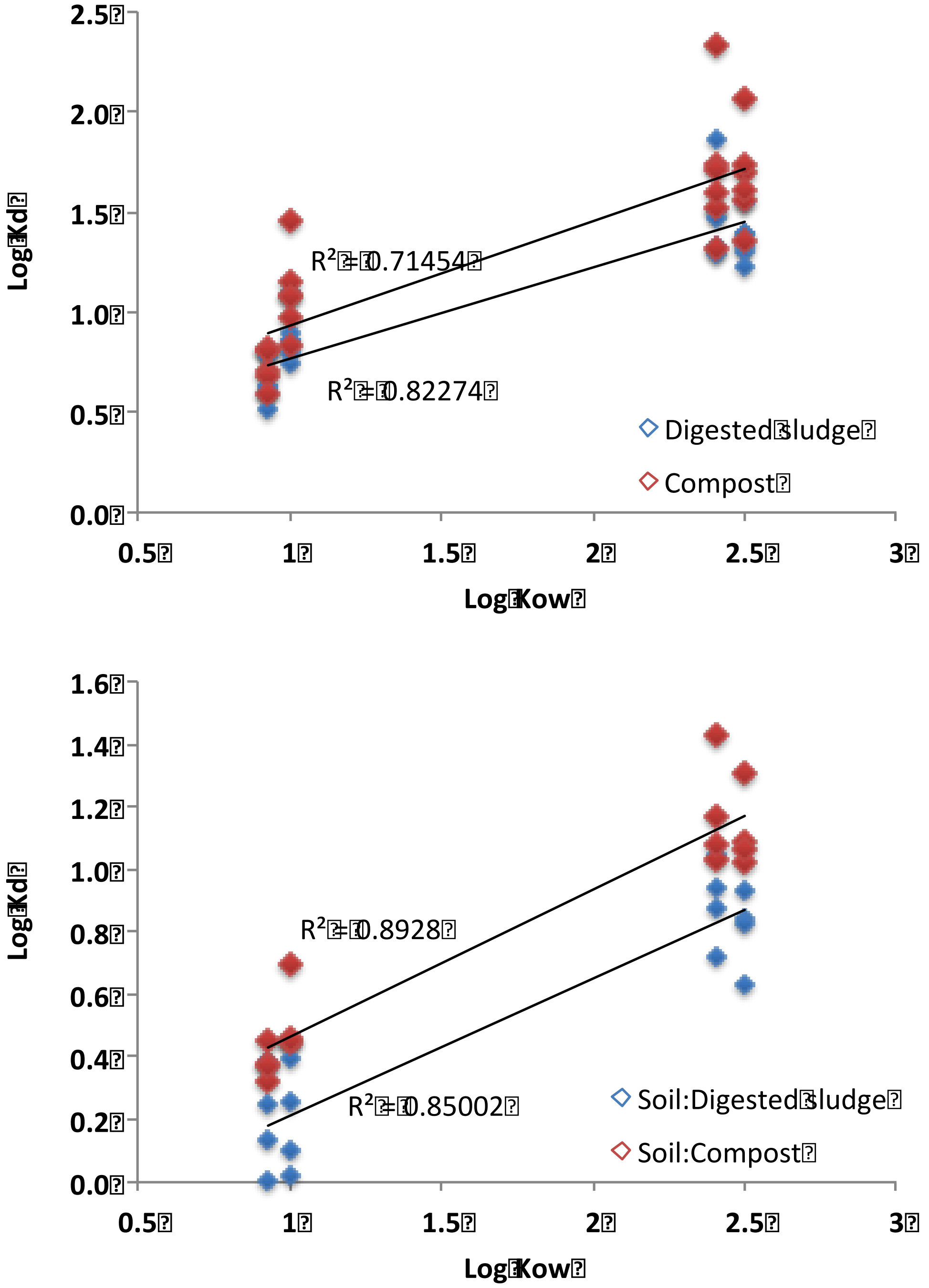

Figure S1. Correlations between log K_d and log K_{ow} for CBZ and its metabolites in sewage sludge (up) and soil mixtures (bottom) (*n*=42).
